# Digital compassion fatigue in nurses: an evolutionary concept analysis

**DOI:** 10.3389/fpubh.2026.1957080

**Published:** 2026-08-31

**Authors:** Xiaobing Wu, Xia Yang

**Affiliations:** Department of Anesthesiology, Shandong Provincial Hospital Affiliated to Shandong First Medical University, Jinan, Shandong, China

**Keywords:** compassion fatigue, concept analysis, digital compassion fatigue, digital empathy, nurses, occupational health, technology-mediated care

## Abstract

Electronic health records, telehealth and home monitoring are changing the psychosocial conditions of nursing work, and digital compassion fatigue in nurses has recently been proposed to describe compassion fatigue that may evolve within technology-mediated care. Direct evidence remains minimal, comprising two concept studies and one commentary, and the boundaries with compassion fatigue, burnout, technostress, moral distress and digital fatigue are unsettled. This study applied Rodgers' evolutionary concept analysis, which, unlike approaches that fix defining attributes at a single point in time, treats concepts as provisional and traces their development across time, disciplines and contexts; to our knowledge it is the first evolutionary analysis of this concept. PubMed, Embase and EBSCOhost (CINAHL Ultimate; Academic Search Premier) were searched to 2 August 2026, identifying 545 records (287 after cross-database de-duplication), supplemented by purposive sampling of adjacent literatures to saturation, citation tracking and hand searching; 38 concept-related papers were included. Five defining attributes are proposed as theoretical propositions rather than established findings: specificity to technology-mediated care; persistent post-encounter emotional exhaustion; compassion withdrawal and relational detachment; cognitive overload during digital interaction; and diminished therapeutic presence and professional efficacy. Each attribute is anchored in the direct conceptual literature and corroborated only by adjacent empirical evidence; proposed antecedents and consequences likewise rest largely on adjacent evidence and are presented as hypotheses rather than causal claims. The principal limitation is the near absence of direct evidence. Digital compassion fatigue is therefore best treated as a provisional working concept, possibly a context-specific subtype of compassion fatigue rather than a new construct, pending tests of discriminant validity. The analysis contributes an attribute-level evidence map, a testable working definition and candidate indicators to guide instrument development and occupational-health-oriented prevention research.

## 1 Introduction

Electronic health records, telehealth and home monitoring are reshaping how nursing work processes information and where the boundaries of clinical interaction lie (1). In technology-mediated care, nurses must simultaneously manage data, interfaces and alerts while recognizing patient suffering and sustaining compassionate care through video, voice, text and monitoring information. A scoping review has shown that digital technologies can extend the reach of compassionate care while also altering the cues and interactional conditions through which compassion is expressed (2); a further systematic scoping review reported that technology may operate by enabling direct expression, fostering self-compassion or supporting interpersonal compassion, but that existing technologies lack comprehensive evaluation of compassion-related dimensions (3). Within this transition, the psychological cost of sustaining compassionate care is beginning to take new forms. From an occupational health perspective, these changes constitute an evolving configuration of psychosocial job demands; candidate concepts describing their human cost therefore require careful scrutiny before being adopted into workforce surveillance or intervention practice.

In 2025, Byrne proposed digital compassion fatigue in nurses (hereafter, digital compassion fatigue) against a background of technostress, describing it as a possible evolution of compassion fatigue among registered nurses working under remote care demands and sustained technological load (4). Abou Hashish and Alnajjar (5) subsequently applied the Walker and Avant approach and identified emotional numbing, persistent emotional exhaustion, compassion withdrawal, cognitive overload, reduced professional efficacy and diminished therapeutic presence as its principal attributes.

The concept nevertheless remains at an early stage of formation. Only two concept studies address it directly, no validated instrument exists, and unclear boundaries carry a twofold risk: ordinary screen fatigue or frustration with system use may be misinterpreted as compassion depletion, and problems properly attributable to organizational and technological design may be over-attributed to individual nurses. The two existing analyses, moreover, leave three problems unresolved (Section 2.2): the absence of operational criteria for differentiating the concept from rival constructs, limited integration with occupational health theory, and a fixed-attribute logic that leaves the concept's ongoing evolution unexamined. Rodgers' evolutionary concept analysis, which emphasizes that concepts change continuously across time, disciplines and contexts, addresses precisely these gaps (6, 7). This study accordingly used that method to examine the evolution, related concepts, defining attributes, antecedents, consequences and empirical referents of digital compassion fatigue, and to propose a working definition for subsequent verification. Given the thinness of direct evidence, the analysis treats digital compassion fatigue throughout as a candidate concept whose status—distinct construct, subtype of compassion fatigue, or redundant relabeling—remains open.

## 2 Background

### 2.1 Origins and evolution of the concept

Compassion fatigue has traditionally described the physical and psychological depletion arising when helpers are repeatedly exposed to the suffering of others and invest in compassionate responses. A nursing concept analysis characterized its manifestations as emotional exhaustion, reduced empathic capacity, numbing and secondary traumatic stress (8). A meta-analysis indicated that compassion satisfaction, burnout and secondary traumatic stress among nurses are generally at moderate levels, with comparatively higher risk in intensive care and high-trauma-exposure settings (9). This tradition was built largely on face-to-face care.

Digital technologies have changed both how patient suffering is presented and the conditions under which nurses respond. Nurses may encounter patient information through video, telephone, text, images, monitoring curves and alerts, yet these interactions commonly lack touch, holistic observation and subtle non-verbal feedback. A rapid review of telecommunication-based interaction found that empathic expression remains achievable by telephone or remote contact, but that the absence of non-verbal cues and touch constitutes an important barrier (10); qualitative work on virtual nursing care showed that nurses compensate for such media constraints by intensifying eye contact, modulating tone, asking proactively and confirming repeatedly (11).

Against this background, the concept was proposed (4) and further specified (5), developing from generic suffering exposure and compassion depletion into a contextual construct combining digital mediation, technological load and depletion of relational resources. In 2026, a peer-reviewed commentary framed digital compassion fatigue as a tangible risk in technology-mediated care and argued that digital empathy must be deliberately designed, taught and protected (12), indicating early uptake within the discipline. Whether this development marks a genuinely distinct construct or a context-specific manifestation of compassion fatigue is examined critically in Section 2.2 and returned to in the Discussion.

### 2.2 Differentiation from related concepts

Alternative terms used interchangeably in the literature include e-compassion fatigue, digital empathy fatigue, online compassion fatigue and technology-mediated compassion fatigue (4, 5). The concept overlaps with traditional compassion fatigue, secondary traumatic stress, burnout, technostress, moral distress and digital fatigue, yet its necessary trigger conditions and core functional impairment differ: digital compassion fatigue is specific to technology-mediated compassionate care and is identified principally through compassion withdrawal, relational detachment or diminished therapeutic presence. Differences between the related concepts are presented in Table 1.

**Table 1:** Comparison of digital compassion fatigue in nurses with related concepts.

Concept	Principal trigger conditions	Core manifestations	Typical contexts	Distinction from digital compassion fatigue
Digital compassion fatigue in nurses	Sustained technology-mediated compassionate care; exposure to digitally presented suffering; technology-mediated emotional labor	Persistent emotional exhaustion; cognitive overload; compassion withdrawal; diminished presence and efficacy	Remote nursing; online consultation; video follow-up; home monitoring	Combines digital mediation with compassionate care
Traditional compassion fatigue	Repeated exposure to patient trauma, suffering or death	Reduced compassionate capacity; emotional numbing; secondary traumatic stress	Emergency, critical care, oncology, palliative care	Digital mediation is not a necessary condition
Secondary traumatic stress	Indirect exposure to the trauma of others through empathic engagement	Trauma-spectrum symptoms: intrusion, avoidance, arousal	Trauma-exposed helping work, including nursing	Symptom profile is trauma-specific and does not require digital mediation
Moral distress	Situational or institutional constraint preventing action one judges ethically right	Moral conflict, guilt, frustration, moral residue	Resource-limited or constrained practice, including digital care	Trigger is ethical constraint rather than mediated exposure to suffering; core impairment is moral rather than compassionate functioning
Burnout	Prolonged workload; insufficient resources; low control; organizational conflict	Emotional exhaustion; cynicism or detachment; reduced professional efficacy	Occupations generally, including nursing roles	Does not require exposure to patient suffering, nor is impaired compassion its core
Technostress	Techno-overload, invasion, complexity, uncertainty and insecurity	Anxiety; frustration; cognitive load; avoidance of use	Electronic health records; medical devices; digital platforms	Can occur without emotional interaction with patients
Digital fatigue	Prolonged screen use; information switching; constant connectivity	Visual fatigue; reduced attention; cognitive exhaustion; social withdrawal	Online work, teaching and digitalized clinical work	Does not necessarily involve patient suffering or compassion withdrawal

Critical comparison indicates that the constructs in Table 1 cannot be separated symptom by symptom: emotional exhaustion, for example, is a shared manifestation of burnout, compassion fatigue, technostress outcomes and digital fatigue alike. Differentiation is therefore configural, resting on the combination of a necessary trigger condition and a core functional impairment rather than on any single symptom. Moral distress illustrates the point: it may co-occur with digital compassion fatigue when technological constraints prevent nurses from providing the care they judge right, yet its trigger is ethical constraint rather than mediated exposure to suffering, and its core impairment concerns moral rather than compassionate functioning.

Appraised critically, the two existing analyses (4, 5) leave three problems unresolved despite their pioneering contribution. First, neither provides an operational rule for excluding rival states: without a necessary-condition structure, any screen-related distress can be labeled digital compassion fatigue, a pattern the screening corpus of the present study illustrates (Section 4.1). Second, antecedents were cataloged descriptively and largely at the individual level, without integration into occupational health theory, leaving prevention implications centered on individual resilience. Third, both adopt the fixed-attribute logic of the Walker and Avant tradition (13), which presumes a conceptual maturity that a two-paper evidence base cannot support and risks premature closure; consistently, neither analysis examined the concept's development over time and across disciplines, which is the central concern of an evolutionary approach (6).

The present analysis responds to these three problems directly: it specifies a necessary trigger condition and discriminating cues (Table 1), embeds antecedents and consequences in the job demands-resources and conservation of resources frameworks, and traces the concept's trajectory from face-to-face compassion fatigue through digital empathy discourse to its current, still-contested usage. On present evidence we treat digital compassion fatigue as a context-specified working concept, plausibly a subtype of compassion fatigue rather than an independent construct, and regard the choice among these interpretations as an empirical question for discriminant-validity research (Section 5.2).

## 3 Methods

### 3.1 Data sources and search strategy

PubMed, Embase and the EBSCOhost platform (CINAHL Ultimate and Academic Search Premier) were used as systematic search databases, with final search dates of 31 July to 2 August 2026. Searches used title and abstract fields without date or language restrictions; complete search strings for each database are provided in Supplementary Table S1, together with line-by-line yields for PubMed and Embase. For the EBSCOhost platform, line-level intermediate counts were not retained at export, and the de-duplicated yield of the primary screening set is reported at platform level. Searching proceeded at two levels with distinct logical functions. The first level was a census of the direct literature: a core concept search combined “digital compassion fatigue” and its alternative terms with a compassion fatigue concept block (compassion fatigue, secondary traumatic stress, compassion satisfaction), a digital context block (digital, telehealth, telenursing, electronic health record, videoconferencing, alarm fatigue and related terms) and nurs^*^, was executed separately in each platform, and identified 545 records (PubMed 176; EBSCOhost 163 after de-duplication of exports; Embase 206). For this direct corpus the aim was completeness, and every eligible record was retained, so no sampling was involved.

The second level addressed the adjacent literatures through purposive sampling, implementing Rodgers' requirement to examine a concept's use across related fields (14): focused searches of eight predefined adjacent domains (digital empathy; technostress in nurses; digital fatigue; empathy and therapeutic relationships in remote care; electronic health record burden; emotional labor in nursing; therapeutic presence and telepresence; and systematic reviews of compassion fatigue in nurses) were conducted in PubMed and sampled purposively, prioritizing evidence syntheses, concept analyses and primary studies of nurses. Because the domains were fixed in advance to keep the process auditable, this procedure constitutes purposive sampling with a saturation-based stopping rule rather than theoretical sampling in the emergent, grounded-theory sense. The aim at this level was conceptual sufficiency rather than exhaustive synthesis; completeness claims are made only for the direct corpus, and the distinction between the two levels is maintained throughout the reporting.

Classic theoretical works (such as the job demands-resources model and conservation of resources theory), methodological and reporting-guidance papers, and three Chinese-language papers reflecting the “Internet Plus Nursing Service” context were added through citation tracking and hand searching; Chinese-language databases were not searched systematically. The three contextual papers were not used to derive attributes: consistent with the evolutionary emphasis on contextual variation, they inform organizational antecedents in one rapidly scaling national telenursing model and flag transferability questions for international application. Only peer-reviewed journal publications were eligible; conference abstracts, trial registry records and preprints were excluded. Papers were included if they addressed digital compassion fatigue directly, or if they were concept analyses, systematic reviews, meta-analyses, qualitative or quantitative studies capable of elucidating its defining attributes, antecedents, consequences, related concepts or empirical referents; papers were excluded if the study population was unrelated to nursing, if they concerned only general patient experience of technology, if the full text was available in neither English nor Chinese, or if bibliographic information was incomplete or duplicated.

Title and abstract screening and full-text checking were conducted independently by two researchers. Disagreements were first addressed by joint re-reading of the record against the written inclusion criteria; where uncertainty persisted, the record was carried forward conservatively to full-text assessment and re-examined once the complete coding frame existed. No disagreement remained unresolved after this procedure. Formal inter-rater agreement statistics were not calculated, which is acknowledged as a limitation; every record-level decision is, however, published in the Supplementary Screening log to permit audit.

After cross-database de-duplication, the 545 identified records yielded 287 unique records for title and abstract screening, of which 267 were excluded (principal reasons: digital terms appearing only incidentally, for example in relation to online survey administration; conference abstracts and trial registry records; populations that were not nurses; digital elements functioning as an intervention delivery mode or research method; and topics unrelated to the concept—counts in Supplementary Figure S1, record-level decisions in the Supplementary Screening log). Of the 20 topically relevant records, five were included (two concept studies, one directly related commentary and two studies of alarm fatigue); the remaining 15 addressed themes already covered by included evidence and were not sampled again.

Saturation for the second level was operationalized as follows: within each adjacent domain, sampling proceeded from evidence syntheses to primary studies and stopped when successive candidate records contributed no new content codeable to attributes, antecedents or consequences. This judgement was made independently by the two researchers for each domain and confirmed by consensus, and the corresponding record-by-record decisions are labeled “relevant - not sampled” in the screening log. The final corpus comprised 38 concept-related papers (two concept studies, one commentary and 35 adjacent papers: two alarm-fatigue studies retained from the systematic core search, 21 sampled purposively across the eight domains, nine added through citation tracking—chiefly theoretical works and evidence syntheses located in the reference lists of included papers—and three Chinese contextual papers added by hand searching), alongside seven methodological and reporting-guidance papers. The paper-by-paper composition, with identification route and domain, is mapped in Supplementary Table S2.

The database set was chosen to combine MEDLINE's biomedical core (via PubMed), Embase's complementary European and allied-health indexing, and CINAHL's nursing-specific coverage—the standard core for nursing reviews—with Academic Search Premier adding multidisciplinary breadth. The marginal yield of the added platforms was small (Embase contributed no additional direct paper; EBSCOhost contributed one directly relevant commentary), suggesting sharply diminishing returns from further databases for so small a direct corpus. Scopus and Web of Science are citation indexes whose journal coverage overlaps the searched sources substantially, and ProQuest primarily indexes dissertations and gray literature, which were ineligible. PsycINFO was not searched; its absence, together with the possibility of publication bias against critical or null discussions of an emerging concept, is acknowledged in the limitations.

### 3.2 Analysis

Rodgers' evolutionary concept analysis holds that concepts are not fixed entities but evolve with context of use and knowledge development (6, 14); the approach avoids prematurely fixing an emerging concept, but advances nursing knowledge only when connected to testable questions, measurement and practice (7). Analysis followed a predefined eight-step workflow (Supplementary Table S3): identification of the concept and surrogate terms; census of the direct literature; two-level screening with logged decisions; purposive sampling of the eight adjacent domains to saturation; verbatim extraction; coding; application of an attribute-retention rule with evidence-status labeling; and derivation of the framework, working definition and hypotheses.

Extraction used a structured matrix in which verbatim segments from each included paper were assigned to six categories: concept use and definition, attributes, antecedents, consequences, related terms, and empirical referents. Segments were open-coded and grouped through constant comparison between direct and adjacent evidence. A candidate defining attribute was retained only if it was anchored in at least one direct conceptual source and corroborated by at least two independent adjacent sources; candidate content lacking direct anchorage was classified as antecedent, consequence or boundary information rather than as a defining attribute. Content supported only by adjacent research was labeled as theoretical inference throughout and never expressed as established causation. Attribute derivation was drafted by one researcher and audited in full by the second against the extraction matrix, with divergences resolved by returning to the source segments.

Two further methodological choices require justification. First, cases: Rodgers advocates identifying real exemplar cases from the literature, but with only two direct concept papers no complete real case was available, so cases were constructed with reference to the strategy of Walker and Avant (13), drawing on the attributes and contexts reported in the included literature; they serve solely as educational illustrations of conceptual boundaries (Section 4.4). Second, quality appraisal of included papers was intentionally omitted: in concept analysis, papers function as data about how a concept is used rather than as estimates of effect, and standard appraisal instruments do not address this purpose (7). Evidential weight was instead managed through the direct/adjacent labeling used throughout and by prioritizing evidence syntheses within adjacent domains; the absence of formal appraisal is acknowledged in the limitations. As no EQUATOR-listed reporting guideline exists for concept analysis, reporting followed the workflow set out above to ensure transparency.

## 4 Overview of the concept

### 4.1 Defining attributes

Drawing on the direct conceptual literature together with adjacent evidence from digital empathy, remote care and technostress, we propose five defining attributes (4, 5). Their evidential basis is mapped attribute by attribute in Table 2, which distinguishes direct conceptual sources from adjacent empirical corroboration; because no primary empirical study of digital compassion fatigue itself yet exists, every attribute has the status of a theoretical proposition. Relative to the six attributes previously identified (5), three adjustments were made, each of which arose inductively from the present analysis rather than by rearrangement alone. First, the elevation of technology-mediated care from background condition to necessary trigger emerged from the screening corpus itself: the single most frequent reason for exclusion (141 of 267 records) was that digital terms appeared only incidentally, which illustrates how readily the concept absorbs any screen-adjacent distress in the absence of a necessary-condition rule. Second, the subsumption of emotional numbing within compassion withdrawal reflects a structural claim—that numbing, shortened enquiry and formulaic response mark points on a single continuum of withdrawal rather than separate attributes—with direct implications for measurement (graded severity items rather than separate subscales). Third, the combination of therapeutic presence and professional efficacy encodes a covariance hypothesis grounded in the telehealth relationship literature (15, 16). What is evolutionary in the result is not the list itself but its status: the attributes are offered as a provisional configuration expected to change as empirical work accumulates (6).

**Table 2:** Evidence status of the proposed defining attributes, antecedent domains and consequence levels, with candidate empirical indicators.

Element	Direct conceptual evidence	Adjacent empirical evidence	Evidence status	Candidate empirical indicators
Defining attributes				
Specificity to technology-mediated care	(4, 5, 12)	(2, 3)	Direct-conceptual, adjacent corroboration; necessary-condition status proposed in this analysis	Proportion of care episodes delivered through digital media; documented responsibility for compassionate response within digital encounters
Persistent post-encounter emotional exhaustion	(4, 5)	(8, 9)	Direct-conceptual, adjacent corroboration; temporal-continuity emphasis proposed here	Post-encounter recovery time; end-of-shift residual distress captured by momentary ratings
Compassion withdrawal and relational detachment	(5)	(10, 11, 17)	Direct-conceptual, adjacent corroboration; continuum structure proposed here	Shortened emotional enquiry; formulaic response frequency; declining personalization of messages (self-report plus interaction records)
Cognitive overload during digital interaction	(4, 5)	(1, 18)	Direct-conceptual, adjacent corroboration	Perceived overload during encounters; task-switching frequency; avoidance of messages and alerts
Diminished therapeutic presence and professional efficacy	(5)	(15, 16)	Direct-conceptual, adjacent corroboration; coupling of the two elements proposed here	Patient-rated presence; nurse-rated efficacy specific to virtual care
Antecedent domains				
Patient- and task-related factors	(5)	(8, 21)	Adjacent-empirical (cross-sectional and qualitative) plus theoretical inference; no concept-specific evidence	—
Technological and media-related factors	(4, 5)	(22–24)	Adjacent-empirical (cross-sectional) plus theoretical inference; alarm-fatigue findings inconsistent	—
Individual factors	(5)	(25–27)	Adjacent-empirical plus theoretical inference	—
Organizational factors	(5)	(28–30)	Adjacent-empirical (meta-analytic for empowerment; qualitative for context) plus theoretical inference	—
Consequence levels				
Nurse level	(5)	(9, 31, 32)	Adjacent-empirical; direction of effect assumed, not demonstrated	—
Nurse–patient relationship level	(5)	(33, 34)	Theoretical inference from empathy-outcome evidence; requires paired designs	—
Organizational level	(5)	(35, 36)	Adjacent-empirical (burnout analog); hypothesis only	—

No element in this table is supported by primary empirical studies of digital compassion fatigue itself; “direct conceptual evidence” denotes the two concept studies and one commentary. Candidate indicators are proposals for future instrument development, not validated measures.

#### 4.1.1 Specificity to technology-mediated care

Digital compassion fatigue arises first and foremost within technology-mediated compassionate care. Technology is not merely a backdrop but the interactional condition determining how suffering is presented, which relational cues are preserved and how nurses express concern. Only when nurses are repeatedly exposed to patient suffering through digital media while carrying responsibility for compassionate response can general technological load convert into digital compassion fatigue. Discomfort arising solely from system failure, screen use or information volume, unaccompanied by impairment of compassionate functioning, does not constitute digital compassion fatigue.

#### 4.1.2 Persistent post-encounter emotional exhaustion

This exhaustion is manifest not only as tiredness during a digital encounter but also as depletion persisting after the interaction has ended. Having closed a consultation interface or completed message replies, nurses may still feel emotionally depleted, irritable or powerless, or find it difficult to recover from a preceding high-emotional-load interaction. What is decisive is not general work fatigue but the temporal continuity between residual emotion and compassionate investment.

#### 4.1.3 Compassion withdrawal and relational detachment

To limit continued depletion, nurses may become less sensitive to digitally presented cues of suffering, shorten emotional enquiry, adopt formulaic responses, avoid complex emotional issues, or reduce affective investment in video and text-based interaction. Research on real-time text-based health consultation indicates that digital empathy requires reciprocal engagement, timely response, authentic expression and individualized information processing; where these requirements are persistently compromised, interaction tends to become mechanical (17). Such compassion withdrawal is an important attribute distinguishing digital compassion fatigue from general screen fatigue, and may further present as relational detachment and emotional numbing.

#### 4.1.4 Cognitive overload during digital interaction

Nurses must simultaneously process clinical information, interface operation, patient emotion, network latency and the absence of communicative cues. Limited attentional resources are repeatedly reallocated between task switching and affective judgement, which may result in reduced attention, difficulty in information processing, decision fatigue and avoidance of messages and alerts; an integrative review of the electronic health record's impact on nurses' cognitive work documents this fragmentation and reallocation of attention (18), and a systematic review links electronic health record-related stress to burnout across hospital clinicians (1). Cognitive overload here is not an independent form of technostress but is interwoven with sustained compassionate response.

#### 4.1.5 Diminished therapeutic presence and professional efficacy

As exhaustion and compassion withdrawal accumulate, nurses may find it difficult to sustain the therapeutic presence through which patients feel seen, understood and supported, and may come to doubt their capacity to deliver high-quality compassionate care in virtual environments. A concept analysis of therapeutic relational connection in telehealth treats the deliberate establishment and use of relational connection as the core process for achieving therapeutic goals (15), and work on telepresence similarly emphasizes the patient's experience of a professional's presence, attention and support (16).

### 4.2 Antecedents

The job demands-resources model and conservation of resources theory offer an integrative account of antecedents: where digital job demands and exposure to patient suffering continuously consume nurses' attentional and emotional resources while technical support, job autonomy, recovery opportunities and leadership support are insufficient, resource depletion accumulates more readily (19, 20). Current evidence suggests that digital compassion fatigue may arise from the joint operation of patient- and task-related, technological and media-related, individual and organizational factors, rather than from a single technological problem or individual characteristic.

**Figure 1:**
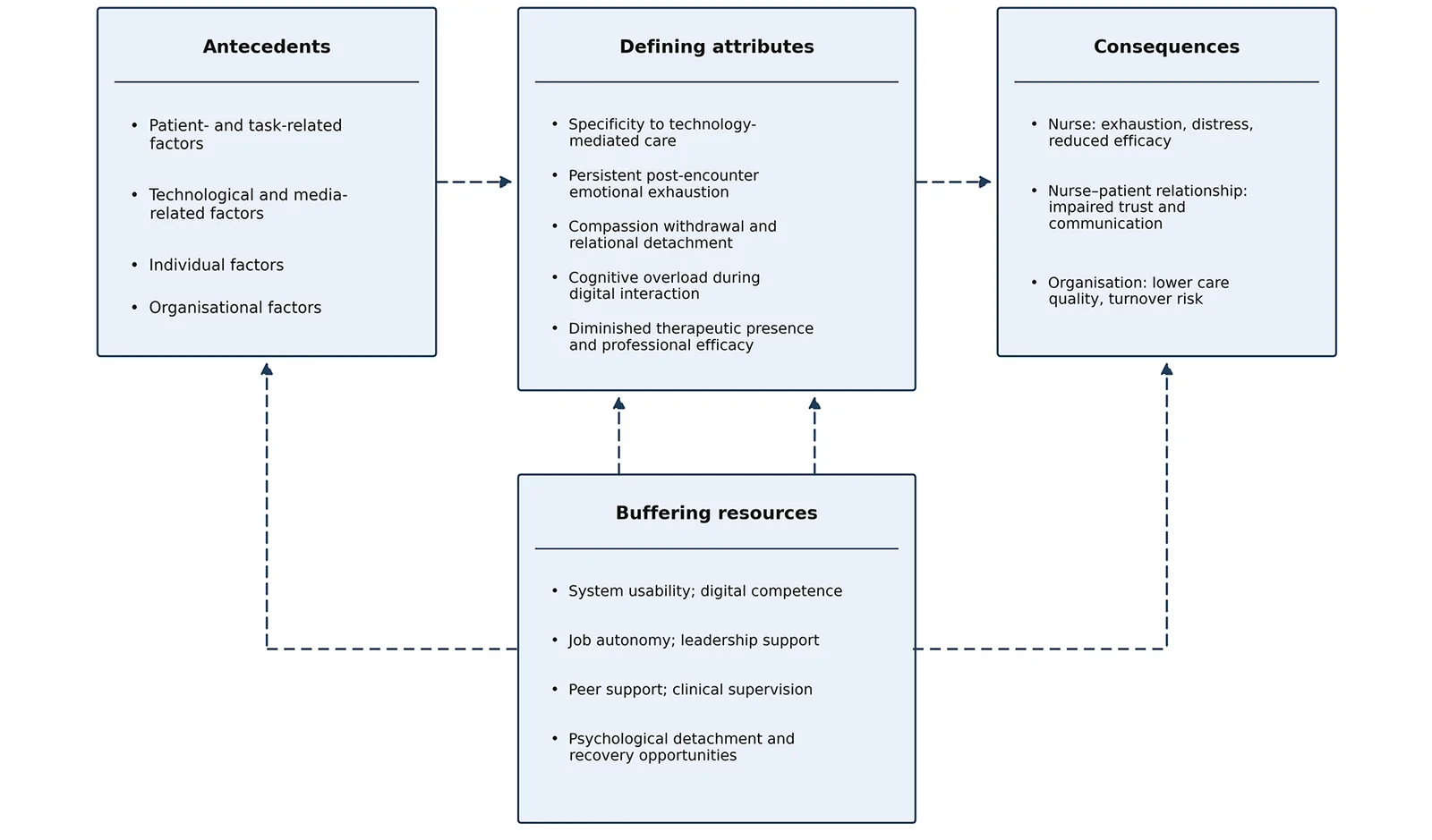
Conceptual framework of digital compassion fatigue in nurses. All arrows are dashed to denote theoretically proposed (hypothesized) pathways: no longitudinal or causal evidence yet exists for the concept itself. The framework serves conceptual explication and is not a causal model or clinical diagnosis.

Attributes and antecedents are related but categorically different, and the framework implies no temporal sequence. The five attributes are co-defining constituents of the state itself, all of which must be present for the concept to apply; antecedents are conditions that precede the state and make it more probable. No causal chain from antecedents to attributes—nor any ordering among the attributes—is asserted; all pathway statements are hypotheses (Figure 1). Within the job demands-resources structure, the four antecedent domains map onto job demands (patient- and task-related exposure; technological and media demands), personal resources and vulnerabilities (individual factors) and job resources (organizational factors), with conservation of resources theory supplying the depletion mechanism: loss spirals become likely when demand-driven resource loss outpaces replenishment (19, 20). The technological and media domain is likewise distinguished from the attribute of cognitive overload: the domain describes external, system-level demand conditions (usability, duplicate entry, alert design), whereas the attribute describes the internal experiential state that arises during care encounters when those demands compete with compassionate engagement.

#### 4.2.1 Patient- and task-related factors

Repeated exposure to patient pain, anxiety, clinical deterioration and family difficulty is an important condition for traditional compassion fatigue (8). In remote follow-up, chronic disease management and home monitoring, nurses must additionally assess and respond to complex needs under conditions of restricted relational cues (21). Case complexity, consecutive consultations and inadequate recovery between encounters may further increase risk, although these factors require prospective verification.

#### 4.2.2 Technological and media-related factors

Poor system usability, duplicate data entry, switching between multiple platforms, network latency, technical failure and insufficient interoperability consume cognitive resources that nurses could otherwise devote to understanding patients. A study of nurses in German hospitals found that technological complexity, overload, lack of technical support and system unreliability were associated with burnout (22). Media differ in the extent to which they preserve non-verbal cues; the more limited the cues, the more nurses must compensate through questioning, confirmation and emotional expression (21). Findings on the relationship between alarm fatigue and compassion fatigue remain inconsistent (23, 24).

#### 4.2.3 Individual factors

Limited digital competence may increase the burden of troubleshooting and the sense of loss of control, while excessive personal distress, emotional over-involvement and unclear professional boundaries may amplify compassionate depletion (5). Nurses must also regulate emotion to meet the expressive requirements of professional digital interaction; a systematic review of emotional labor in mental health nursing showed that emotional labor is interrelated with emotional exhaustion, burnout and turnover risk (25). Conversely, greater psychological resilience, clear boundaries, active coping and psychological detachment after work may facilitate resource recovery (26, 27).

#### 4.2.4 Organizational factors

Inadequate staffing, excessive digital consultation volume, insufficient training time, absent technical support and clinical supervision for remote care, performance appraisal weighted toward service volume rather than relational quality, and unclear boundaries around out-of-hours contact may all compress opportunities for recovery. A systematic review and meta-analysis found that structural and psychological empowerment were negatively associated with emotional exhaustion and depersonalization among nurses, suggesting that autonomy, resources and managerial support may be protective (28). Chinese studies of “Internet Plus Nursing Service” report that nurses' practice experience involves insecurity, role conflict, insufficient support, risks associated with unfamiliar environments and pressure in managing emergencies (29, 30). Although these studies did not measure digital compassion fatigue directly, they provide contextual evidence for its organizational antecedents.

### 4.3 Consequences

Existing direct concept research groups the potential consequences of digital compassion fatigue at the nurse, nurse–patient relationship and organizational levels (5). The three levels are summarized, with their evidence status, in Table 2; none is supported by longitudinal or concept-specific evidence, directionality is assumed rather than demonstrated, and the following should therefore be read as hypotheses informed by adjacent evidence.

#### 4.3.1 Nurse level

Potential manifestations at nurse level include persistent exhaustion, emotional numbing, reduced attention, avoidance of digital care tasks and diminished professional efficacy. Traditional compassion fatigue research suggests possible co-occurrence with burnout and secondary traumatic stress (9), while research on technostress and digital fatigue points to adjacent outcomes such as emotional exhaustion, psychological distress and impaired work motivation (31, 32). The latter two categories constitute indirect support only, and the direction of causation cannot be determined from them.

#### 4.3.2 Nurse–patient relationship level

Where nurses reduce emotional enquiry, adopt mechanical responses or avoid complex emotional issues, patients' sense of attention and support may be weakened. An interprofessional systematic review found that clinician empathy is associated with patient experience and care outcomes and is influenced by modifiable factors including organizational context (33); a systematic review of randomized trials further indicated that enhancing practitioner empathic expression can improve patient satisfaction (34). This evidence supports the theoretical plausibility of relational consequences but does not constitute direct causal evidence for digital compassion fatigue, and requires verification through paired nurse–patient designs.

#### 4.3.3 Organizational level

Long-term accumulation of digital compassion fatigue may affect care quality and increase turnover risk. A systematic review and meta-analysis of nurse burnout reported associations with poorer safety culture, more adverse events, lower patient satisfaction and lower quality of care (35); among nurses, digital fatigue has been linked to job satisfaction and work motivation (32), and digital stress to emotional exhaustion and work-privacy conflict (36). These findings provide indirect support only for organizational consequences; missed nursing care, error, sick leave, team communication burden and the sustainability of digital nursing services should still be regarded as hypotheses awaiting testing.

### 4.4 Cases

The following constructed cases are educational illustrations of the attribute configuration and its boundaries; they are hypothetical, contribute no empirical evidence, and do not validate the concept (construction rationale in Section 3.2).

#### 4.4.1 Model case

Nurse A has worked in remote oncology follow-up for 8 months, completing consecutive video reviews, text consultations and alert handling daily. She increasingly feels mentally blank when patients express pain or fear, remains emotionally depleted after work, and has begun shortening follow-up questions, using fixed phrasing and avoiding emotive topics. She describes “only completing tasks on a screen,” struggles to make patients feel attended to, and doubts her capacity to provide warm care. All five attributes are present.

#### 4.4.2 Borderline case

Nurse B developed eye strain, operational frustration and data-entry pressure after a new electronic health record was introduced, yet continued to identify and respond patiently to patient emotion during remote follow-up and recovered quickly once training and system optimization were provided. Technostress and digital fatigue are present, but compassion withdrawal, relational detachment and diminished presence are absent; the case is not digital compassion fatigue.

#### 4.4.3 Contrary case

Nurse C works in a team that adjusts remote consultation volume to case complexity, schedules transition time between emotionally demanding encounters, and provides immediate technical support and regular supervision. She conveys attention through tone and paraphrasing, recovers through peer debriefing and detachment after work, and sustains presence and efficacy; no core attribute is present.

### 4.5 Empirical referents and measurement

As at the last search date (2 August 2026), no psychometrically validated instrument specific to digital compassion fatigue had been identified. Pending the development of a specific tool, composite assessment may be used: professional quality of life instruments can capture compassion satisfaction, burnout and secondary traumatic stress (9), and technostress instruments can assess technological overload, constant connectivity and cognitive exhaustion (31). A concept analysis of digital empathy can inform interview dimensions (37), and the Digital Communication Empathy Scale has demonstrated sound preliminary psychometric performance (38); however, it was not developed for nurses and does not measure compassion fatigue, and therefore cannot substitute for a specific instrument. Semi-structured interviews, work diaries or ecological momentary assessment may additionally be used to identify compassion withdrawal, relational detachment and residual emotion after care encounters. Electronic health record usage time, message volume, video consultation volume and alert frequency can serve only as exposure indicators and cannot by themselves establish digital compassion fatigue.

A future instrument should operationalize the candidate indicators proposed in Table 2, spanning exposure to digitally presented suffering, technology-mediated emotional labor, cognitive overload during digital interaction, persistent post-encounter emotional exhaustion, compassion withdrawal, and diminished therapeutic presence or professional efficacy, and should be developed sequentially through item generation from nurse interviews and expert consultation, content validity testing, structural evaluation, and assessment of reliability and measurement invariance (39). Because the central risk is that existing constructs are simply renamed, discriminant validity should be examined explicitly: multitrait-multimethod designs incorporating professional quality of life and technostress measures; competing confirmatory factor models (a single compassion fatigue factor, vs. a correlated digital-specific factor, vs. a bifactor structure); and incremental validity of digital-specific scores in predicting digital-care outcomes beyond generic compassion fatigue. Known-groups comparisons across roles with high and low digital exposure offer a further preliminary test. Should digital-specific variance prove negligible in such designs—a single compassion fatigue factor fitting no worse than models containing a digital-specific factor, and digital-specific scores adding no incremental prediction—the term ought to be retired as a distinct construct label, with “digital” retained only as a description of the care setting. This falsification condition operationally separates the redundancy reading from the subtype reading discussed in Section 5.2, which predicts the opposite pattern: a correlated digital-specific factor carrying incremental validity within the compassion fatigue hierarchy.

### 4.6 Working definition and conceptual framework

Synthesizing the above analysis, we define digital compassion fatigue in nurses provisionally as a state in which nurses, in the course of sustained technology-mediated compassionate care and through repeated exposure to digitally presented patient suffering, and under the influence of insufficient relational cues, technological load and constrained recovery resources, progressively develop persistent post-encounter emotional exhaustion, cognitive overload during digital interaction, compassion withdrawal and relational detachment, and diminished therapeutic presence and professional efficacy. The definition is intended to guide research and risk recognition and does not constitute an independent clinical diagnosis. The conceptual framework is presented in Figure 1.

## 5 Discussion

### 5.1 Significance and boundaries of the analysis

This study used an evolutionary perspective to reorganize the context, attributes and evidential boundaries of digital compassion fatigue (6). The core of the concept is not “feeling tired after using digital technology” but impairment of emotional and relational functioning within technology-mediated compassionate care (5). Technostress and digital fatigue may act as antecedents or co-occurring states; only when persistent exhaustion is further accompanied by compassion withdrawal, relational detachment or diminished therapeutic presence does the presentation correspond more closely to digital compassion fatigue. This differentiation both avoids psychologizing ordinary problems of system use and helps to reduce measurement confounding with burnout and traditional compassion fatigue.

At the same time, digital compassion fatigue should not at present be regarded as an independent syndrome with stable boundaries. The two direct studies are conceptual discussions or concept analyses (4, 5) and the recent commentary is an opinion piece (12); no evidence exists regarding prevalence, diagnostic thresholds, temporal ordering or predictive validity. The five defining attributes and the working definition proposed here should therefore be treated as testable hypotheses rather than settled conclusions.

### 5.2 Distinct construct, subtype, or contextual label? Alternative interpretations

Three readings of the present evidence are possible, and the analysis cannot yet adjudicate among them. Digital compassion fatigue may be a genuinely distinct construct; it may be a context-specific subtype of compassion fatigue; or it may be a redundant relabeling of existing constructs in digital dress. Comparison with the established structure of compassion fatigue (8) argues against full independence: the shared core of exhaustion and diminished empathic capacity is unmistakable. Yet three elements resist reduction to a mere label—the necessary contextual condition, the interweaving of cognitive overload with compassionate response, and the mediated character of therapeutic presence—which argues against pure redundancy. The most defensible current position is the middle one: digital compassion fatigue as a candidate context-specified subtype of compassion fatigue, retained as a working concept because it directs attention to modifiable technological and organizational conditions that generic compassion fatigue obscures. The choice among the three readings is ultimately empirical and will be settled by the discriminant-validity programme outlined in Section 4.5, which separates them operationally: full independence predicts separable factors with distinct antecedent profiles; the subtype reading predicts a correlated digital-specific factor with incremental validity nested within compassion fatigue; and redundancy predicts that a single compassion fatigue factor suffices—the falsification condition stated there.

### 5.3 Implications for research

First, context-specific conceptual validation should be undertaken. Nurses working in internet hospitals, online consultation, remote follow-up, home monitoring and transitional care could be interviewed to examine whether the five defining attributes are recognizable in Chinese digital nursing contexts, and to compare differences across media, roles and patient groups. Second, a specific instrument should be developed with priority given to testing discriminant validity, so that technostress, general digital fatigue and burnout are not simply renamed as digital compassion fatigue; instrument development should proceed sequentially through item generation, content validity, structural construction and evaluation of reliability and validity (39). Third, pre-post shift measurement, ecological momentary assessment, multicenter prospective cohorts and paired nurse–patient designs should be used to test the temporal pathway from digital job demands and suffering exposure, through technology-mediated emotional labor and resource depletion with inadequate recovery, to digital compassion fatigue and its relational and organizational consequences. Ecological momentary assessment can reduce recall bias and capture short-term variation in real-world contexts (40). Objective logs should serve only as exposure indicators and should observe the principle of data minimization and privacy protection.

In addition, reporting standards for concept analysis in nursing are emerging, with a recent systematic review proposing preferred reporting items on the basis of 111 concept analyses (41); subsequent conceptual validation and instrument development studies would benefit from a unified reporting framework, and from testing the cross-context stability of the five defining attributes across different countries and care systems so that the concept is not fixed by a single context.

### 5.4 Implications for practice and workforce management

The implications below are graded by evidential basis: none has been tested against digital compassion fatigue itself, and each is either an extrapolation from adjacent empirical evidence or expert opinion. Organizations should treat them as candidate measures for local evaluation rather than proven interventions.

Prevention of digital compassion fatigue cannot rely on individual resilience training alone; as recently argued, empathy in technology-mediated care must be deliberately designed, taught and protected (12). At the organizational level, healthcare institutions could give priority to reducing avoidable digital job demands, including duplicate data entry, non-actionable alerts, switching between multiple platforms and unreasonable expectations of constant availability; research on technostress in nurses shows that technological complexity, overload, insufficient support and system unreliability are associated with burnout (22). Reducing such avoidable demands is an extrapolation from adjacent empirical evidence. Determining remote consultation volume according to case complexity and emotional care needs, and scheduling transition time between high-emotional-load encounters, are at present expert opinion.

At the level of training and support, curricula could extend beyond software operation to encompass digital empathy, telepresence, breaking bad news online, handling silence, closing virtual relationships and professional boundaries; an umbrella review of empathy, compassion and person-centered communication training supports longitudinal sequenced learning, feedback, reflection and experiential methods (42). The value of longitudinal, experiential training formats is extrapolated from adjacent evidence on empathy training generally; the digital-specific content proposed here is expert opinion. Managers could further provide immediate technical support, peer debriefing, professional supervision and non-punitive psychological support; a meta-analysis of empowerment and burnout in nurses suggests that autonomy, access to resources and managerial support may be protective (28).

Existing interventions for compassion fatigue include psychoeducation, mindfulness, resilience training, reflective practice and peer support. Systematic reviews suggest that such measures may improve compassion fatigue, burnout and secondary traumatic stress, although protocols and methodological quality are heterogeneous (43, 44). An overview of systematic reviews has meanwhile shown that organization-level interventions addressing working time, work organization and the psychosocial work environment can improve outcomes including burnout (45). None of these findings can be extrapolated directly to digital compassion fatigue; future work should compare the independent and combined effects of technological and workflow optimization, rostering and recovery design, training in digital compassionate care, peer supervision and integrated programmes.

### 5.5 Limitations

This study has substantial limitations. First, direct evidence is confined to two concept studies (4, 5) and one opinion commentary (12), so attributes, antecedents and consequences depend heavily on adjacent literatures from digital empathy, technostress, digital fatigue, remote care and traditional compassion fatigue; the analysis provides no empirical validation of its own, and the framework is untested. Second, systematic searching covered PubMed, Embase and the EBSCOhost platform but not PsycINFO, Scopus, Web of Science, ProQuest or Chinese-language databases, and Chinese-language papers entered only through citation tracking and hand searching, so database and language coverage is limited. Third, the purposive sampling of adjacent evidence, although logged record by record, introduces selection risk, and no formal quality appraisal of included papers was undertaken (Section 3.2).

Fourth, publication bias may inflate the apparent uptake of the concept, since critical or null discussions of an emerging construct are less likely to be published (Section 3.1). Fifth, cultural generalizability is limited: the two concept studies arise from single national contexts, the contextual lens added here is Chinese, and the stability of the proposed attributes across care systems and cultures is unknown. Sixth, the constructed cases are educational illustrations only. Searches were completed on 2 August 2026, and concept usage may change as digital health practice develops; the working definition therefore requires continuing testing and revision through subsequent empirical research.

## 6 Conclusion

Digital compassion fatigue in nurses is a provisional working concept, not an established nursing construct. On current evidence it is best understood as a candidate, context-specific form of compassion fatigue arising within technology-mediated care, whose distinctness from existing constructs remains to be demonstrated. Its five proposed defining attributes—specificity to technology-mediated care, persistent post-encounter emotional exhaustion, compassion withdrawal and relational detachment, cognitive overload during digital interaction, and diminished therapeutic presence and professional efficacy—constitute testable hypotheses rather than settled findings, as do the antecedent and consequence pathways proposed around them. Current evidence cannot support an independent diagnosis or establish prevalence, and the definition offered here is provisional pending empirical validation. Future work should test the concept through context-specific qualitative research, instrument development with explicit discriminant-validity criteria, longitudinal examination of mechanisms and multilevel intervention studies, allowing the concept either to mature into a verifiable and modifiable construct or to be folded back into compassion fatigue with a contextual qualifier.

## Data Availability

The original contributions presented in the study are included in the article/Supplementary material, further inquiries can be directed to the corresponding author.
